# Reversal of Apixaban Induced Alterations in Hemostasis by Different Coagulation Factor Concentrates: Significance of Studies *In Vitro* with Circulating Human Blood

**DOI:** 10.1371/journal.pone.0078696

**Published:** 2013-11-11

**Authors:** Gines Escolar, Victor Fernandez-Gallego, Eduardo Arellano-Rodrigo, Jaume Roquer, Joan Carles Reverter, Victoria Veronica Sanz, Patricia Molina, Irene Lopez-Vilchez, Maribel Diaz-Ricart, Ana Maria Galan

**Affiliations:** 1 Department of Hemotherapy-Hemostasis, Hospital Clinic, Centre de Diagnostic Biomedic, Institut d'Investigacions Biomediques August Pi i Sunyer, Universitat de Barcelona, Barcelona, Spain; 2 Bristol-Myers Squibb, Madrid, Spain; 3 Department of Neurology, Hospital Universitari del Mar, Parc de Salut Mar, Barcelona, Spain; National Cerebral and Cardiovascular Center, Japan

## Abstract

Apixaban is a new oral anticoagulant with a specific inhibitory action on FXa. No information is available on the reversal of the antihemostatic action of apixaban in experimental or clinical settings. We have evaluated the effectiveness of different factor concentrates at reversing modifications of hemostatic mechanisms induced by moderately elevated concentrations of apixaban (200 ng/ml) added *in vitro* to blood from healthy donors (n = 10). Effects on thrombin generation (TG) and thromboelastometry (TEM) parameters were assessed. Modifications in platelet adhesive, aggregating and procoagulant activities were evaluated in studies with blood circulating through damaged vascular surfaces, at a shear rate of 600 s^−1^. The potential of prothrombin complex concentrates (PCCs; 50 IU/kg), activated prothrombin complex concentrates (aPCCs; 75 IU/kg), or activated recombinant factor VII (rFVIIa; 270 μg/kg), at reversing the antihemostatic actions of apixaban, were investigated. Apixaban interfered with TG kinetics. Delayed lag phase, prolonged time to peak and reduced peak values, were improved by the different concentrates, though modifications in TG patterns were diversely affected depending on the activating reagents. Apixaban significantly prolonged clotting times (CTs) in TEM studies. Prolongations in CTs were corrected by the different concentrates with variable efficacies (rFVIIa≥aPCC>PCC). Apixaban significantly reduced fibrin and platelet interactions with damaged vascular surfaces in perfusion studies (p<0.05 and p<0.01, respectively). Impairments in fibrin formation were normalized by the different concentrates. Only rFVIIa significantly restored levels of platelet deposition. Alterations in hemostasis induced by apixaban were variably compensated by the different factor concentrates investigated. However, effects of these concentrates were not homogeneous in all the tests, with PCCs showing more efficacy in TG, and rFVIIa being more effective on TEM and perfusion studies. Our results indicate that rFVIIa, PCCs and aPCCs have the potential to restore platelet and fibrin components of the hemostasis previously altered by apixaban.

## Introduction

Despite the better safety profile of newly developed oral anticoagulants, bleeding is still a safety issue and reversal of their pro-hemorrhagic effects for emergency procedures remains unsolved [1]. The lack of effective antidotes to rapidly reverse the anticoagulant action of new oral anticoagulants is becoming a reasonable concern for their expected short-term and future developments [2]–[4]. Although there is a gradually increasing emergence of guidelines and expert opinions on how to proceed for elective and emergency invasive procedures in patients treated with the new oral anticoagulants [5]–[9], there is little evidence to specifically guide the management of the severely bleeding anticoagulated patient.

Based on the experience in animal models, activated or non-activated prothrombin complex concentrates (aPCCs, PCCs) or rFVIIa have been proposed as alternatives to counteract the antihemostatic action of the new anticoagulant therapies [10]–[15]. Two studies have specifically evaluated the potential of coagulation factor concentrates to reverse the anticoagulant effect of rivaroxaban and dabigatran in healthy volunteers [16], [17]. Results of the earlier study revealed that PCCs completely reversed the alterations in the laboratory parameters engendered by rivaroxaban in healthy subjects, but had no effect on the alterations induced by dabigatran [16]. Results of the later study, indicate that PCCs may also improve thrombin generation parameters after dabigatran administration and suggest that lower doses of aPCC may potentially reverse the alterations in coagulation tests caused by rivaroxaban and dabigatran [17]. However, an experimental study has raised some concerns on possible discrepancies between correction of alterations in laboratory parameters and control of bleeding. Godier and cols [18] evaluated the ability of rFVIIa and PCC to reverse the effects of rivaroxaban in a rabbit model of hepatosplenic bleeding. Both rFVIIa and PCC partially improved laboratory parameters, but surprisingly none of these factors reduced rivaroxaban induced-bleeding in their experimental model. It is possible that current laboratory tests applied to the evaluation of specific coagulation pathways may have a poor correlation with clinical bleeding occurring in blood circulating through damaged vessels.

Apixaban is a new oral anticoagulant with a selective inhibitory action on FXa [19]. Despite the safety and efficacy of apixaban demonstrated in clinical trials [20]. There is limited information on possible strategies for the reversal of its antithrombotic effects in patients presenting with medical or surgical emergencies. It has been recently reported that rFVIIa, at very elevated concentrations could partially reverse the inhibitory effect of apixaban in an *in vitro* thrombin generation assay [21]. As previously mentioned for rivaroxaban, there are reasonable doubts on how modifications in thrombin generation assays correlate with the risk of bleeding or its control in the clinical situation [16], [18]. Investigations of the mechanisms involved in the antithrombotic action of apixaban preventing formation of occlusive thrombi at the level of the damaged vasculature and reversal of its antihemostatic action are difficult to perform in patients included in clinical trials.

In the present studies, we have explored the effects of concentrations of apixaban moderately above those achieved at C_max_ after standard approved dosage [22] on platelet and coagulation mechanisms of hemostasis in humans. Moreover, our studies were designed to evaluate the effectiveness of different factor concentrates at reversing the alterations of hemostatic mechanism induced by apixaban. To accomplish these objectives a series of laboratory tests were applied that evaluated modifications in the activation of basic mechanisms of coagulation involved in thrombin generation and thrombus formation. Special attention was paid to the assessment of modifications on the interactions of platelet and fibrin deposition on damaged subendothelium in an *in vitro* thrombosis model with circulating human blood.

## Materials and Methods

### Ethics statement

The investigation conforms with the Directive 2010/63/EU of the European Parliament on the protection of animals used for scientific purposes; and with the principles outlined in the Declaration of Helsinki. This study has been approved by the Hospital Clinic Ethical Committee of Clinical Investigation (registry: 2011/6837). The protocol to isolate rabbit aortas, to be used as thrombogenic substrata, was also approved by the Animal Ethical Committee of the University of Barcelona (number DAAM: 6632).

### Participants, blood sampling and routine laboratory determinations

The study group consisted on 10 healthy volunteers (6 male and 4 female) ages ranging from 22 to 42 years, who agreed to donate blood samples after written informed consent. Individuals who had received acetylsalicylic acid, non-steroidal anti-inflammatory or antiplatelet drugs within 7 days before blood sampling were excluded. Blood samples were collected into tubes (BD Vacutainer, Franklin Lakes, USA) containing citrate (final concentration of 13 mM). Normality of routine hematological parameters: platelet count (normal range, 130–400×10^9^/L), hematocrit (normal range, 0.36–0.51 L/L), WBC count (normal range, 4–11×10^9^/L) were confirmed in an Advia 2120 Hematology System. Similarly, the normality of PT (normal range 11.8–13.9 s), aPTT (normal range 25–30 s) and fibrinogen levels (normal range 1.5–4.5 g/L) was confirmed in a BCS ^TM^ XP system (Siemens, Deerfield, IL, USA).

### Experimental design

Whole blood samples were used to evaluate modifications in: 1) viscoelastic parameters of clot formation in whole blood using by thromboelastometry (ROTEM, Tem International GmbH, Germany); 2) dynamics of thrombin generation in plasma using the fluorogenic assay Technothrombin TGA (Technoclone GmBH, Austria); and 3) perfusion studies with whole blood circulated through damaged vascular segments at an intermediate shear rate of 600 s^−1^, equivalent to that found in medium sized cerebral arteries.

Apixaban (provided by Bristol Myers Squibb Company, USA) was initially dissolved in ethanol, and subsequently diluted in saline. Aliquots of apixaban dilutions were added to blood samples to achieve a concentration equivalent to 200 ng/mL; a concentration twice the average Cmax reached in patients subjected to treatment with the standard dose indicated for the prevention of thrombotic complications in patients with atrial fibrillation [22]. A similar volume of the diluent was added to the control studies.

Coagulation factor concentrates, were tested for their ability to reverse the anticoagulant action of apixaban at doses approved in the prescribing information for each concentrate: rFVIIa: Novoseven®, 270 µg/kg (NovoNordisk, Bagsvaerd, Denmark); aPCC, Feiba® 75 U/kg (Baxter); and PCC; and Beriplex® 50 IU/kg (CSL Behring GmbH, Marburg, Germany). Doses of these concentrates added to blood were calculated assuming that an standard adult weighing 70 kg will have a blood volume of 4900 mL. Aliquots of the different concentrates were spiked in the blood samples to evaluate their potential corrective effect on the different laboratory tests.

### Thrombin generation assay (TGA)

Thrombin generation (TG) was evaluated in citrated platelet poor plasma (PPP) samples with the fluorogenic assay from Technothrombin TGA^TM^ (Technoclone GmBH, Austria) [23], [24]. The activation of the coagulation system was triggered by two different reagents, RCL and RD. RCL consists of low concentration micelles of negatively charged phospholipids containing recombinant human tissue factor (rTF; 7.2 pM final concentration) and CaCl_2_ triggering thrombin generation through the activation of the extrinsic pathway. The RD activating principle contains negatively charged phospholipids and CaCl_2_ triggering thrombin generation through the intrinsic pathway. Fluorescence generated was evaluated at a wavelength of 390 nm/450 nm (excitation/emission) and TG parameters calculated using the Thermo Fluoroskan Ascent Software (Technoclone GmbH). Main parameters used in our studies were lag phase, maximum thrombin peak (nM) and the time to achieve this peak [25].

### Thromboelastometry studies

We investigated the dynamic thrombelastography of whole blood coagulation, using the ROTEM Thromboelastometry Analyser (PentapharmGmbH, München, Germany) [26]. We focused on the analysis of the exTEM test (Rotem Thromboelastometry, Biometa, Spain) in citrated blood, recalcified with 6 mM calcium and using tissue factor as activator. Dynamics of clot formation were followed for 45 min. Clotting time (CT), the time (sec) elapsed from the start until the amplitude of the forming clot reaches 2 mm; clot formation time (CFT), the time (sec) for the tracing reaching 20 mm of amplitude; and maximum clot firmness (MCF) the maximum amplitude of the tracing reached (in mm), were assessed for the purpose of our studies [25].

### Perfusion studies

Aliquots of blood were perfused through annular chambers exposing damaged vascular segments, as thrombogenic substrata. Aortas were extracted from young female New Zealand rabbits (2.8–3.0 Kg) previously euthanized according to protocols approved by the Animal Ethical Committee of the University of Barcelona (number DAAM: 6632). Endothelial cells were removed by exposure to α-chymotrypsin, and vessels were cleaned, everted, cut into segments and maintained in PBS.as previously described [27], [28]. Perfusions studies were performed at a shear rate of 600 s^−1^, for 10 min. Citrate-anticoagulated blood was mixed with a CaCl_2_/MgCl_2_, buffer, before it entered the flow chamber, to achieve physiologic Ca^2+^ and Mg^2+^ of approximately 2 mM in the circulating blood. Perfused vessels were rinsed with 0,15 M PBS, fixed with 2.5% glutaraldehyde in 0.15 M PBS) at 4°C for 24 h and processed histologically for further morphometric evaluation. Fibrin deposition and platelet interactions were evaluated by light microscopy in histological semi-thin cross-sections of the perfused vessels. A specifically developed software, automatically classifies and quantifies platelet and fibrin coverage present in 20 randomly chosen microscopic fields in non-adjacent sections [29]. Overall platelet interactions with the exposed vascular surfaces were evaluated as a percentage of total surface of the vessel covered by platelets (% CS). Presence of larger platelet masses (aggregates of more than 5 µm in height) were also expressed as a percentage of the surface vessel (% Agg). Similarly, the presence of fibrin in the same microscopic fields was also morphometrically quantified and expressed as a percentage of fibrin coverage (% F) and mean area of fibrin masses deposited on the subendothelium (F Area as µm^2^). For detailed information on this methodology, refer to *Methods S1, Perfusion technique.*

### Statistics

Data are expressed as mean ± standard error of the mean (SEM) derived from at least 10 independent experiments. Statistical analysis was performed with raw data using ANOVA after Wilcoxon-Mann-Whitney test for Gaussian distribution. The SPSS statistical package 17.0.0 (SPSS Inc, Chicago, IL) was used for all analyses. Initial levels of statistical significance were established at p<0.05.

## Results

### Influence on thrombin generation

**Figure 1:**
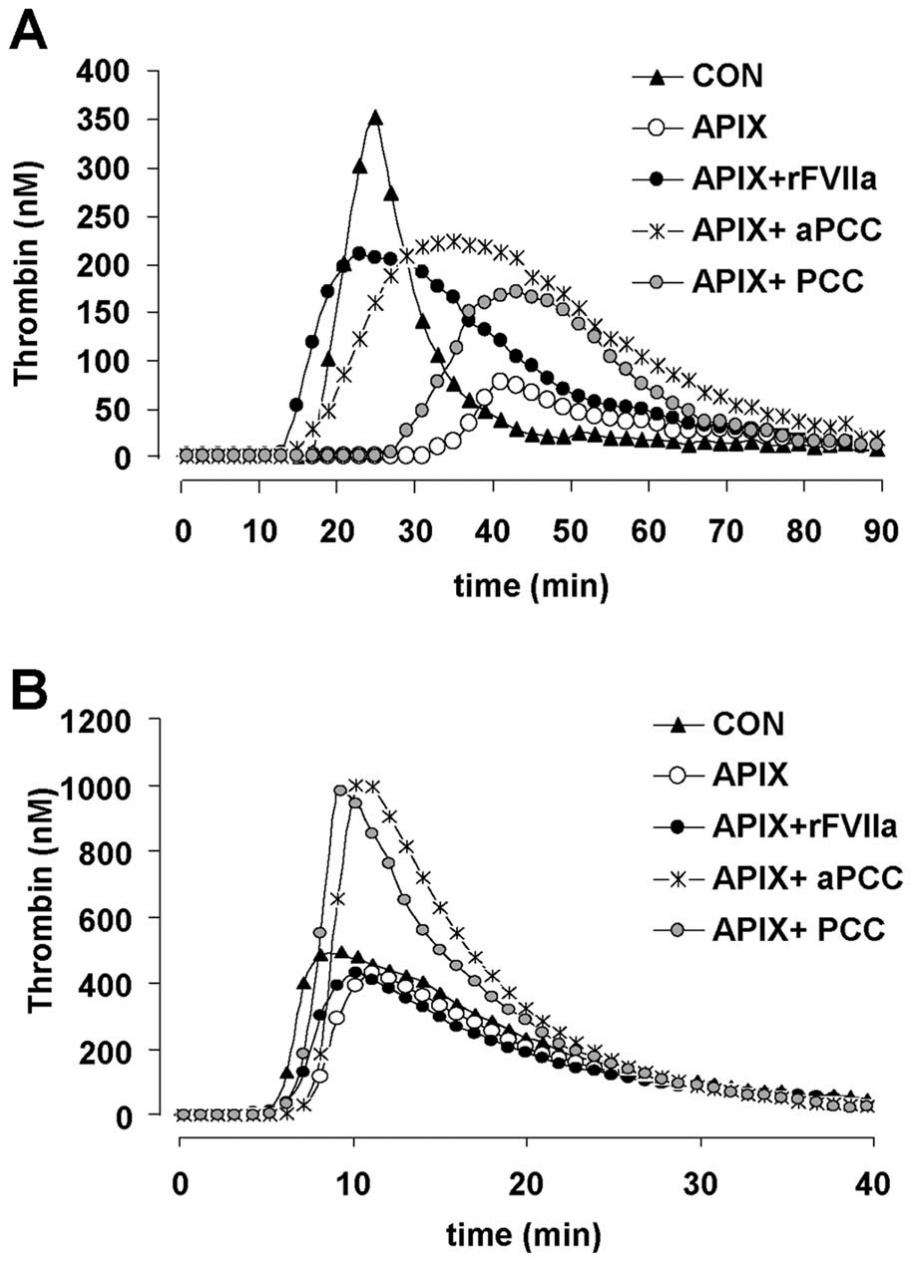
Thrombin generation kinetics in recalcified plasma activated with phospholipids and tissue factor or phospholipid micelles. Representative thrombograms showing the kinetics of thrombin generation in experiments performed triggering thrombin generation in recalcified platelet poor plasma with (**A**) phospholipids and tissue factor or (**B**) phospholipid micelles. Apixaban 200 ng/mL (open circles) delayed and reduced thrombin generation kinetics in plasma. Effects of apixaban on thrombin generation were very evident when activation of the extrinsic coagulation pathway was initiated by tissue factor (**A**) and milder when activation was initiated through the intrinsic coagulation pathway by phospholipids (**B**). All the coagulation factor concentrates improved the alterations in thrombin generation induced by apixaban, with rFVIIa being more efficacious than PCCs or aPCCs at restoring TG altered by apixaban when TG was initiated by tissue factor (**A**) and PCCs and aPCCs dramatically enhancing TG peaks above the levels observed in baseline control experiments when activation was initiated by phospholipids (**B**). Tracings represent mean values form 10 independent experiments. Refer to Tables 1 and 2 for detailed statistics.

As shown in Figure 1a and Table 1, apixaban at 200 ng/mL caused evident alterations in the kinetics of TG initiated by tissue factor and phospholipids (RCL reagent). Alterations induced by apixaban were characterized by a statistically significant delay in the lag phase (34.3±5.5 min vs. 15.5±2.3 min in control experiments; p<0.01), and a dramatic reduction in the maximum thrombin peak achieved during TG (75,8±23 nM vs. 349.7±30 nM in control experiments; p<0.01). Recombinant FVIIa and aPCCs partially restored the alterations in the kinetics of TG caused by apixaban, reducing the prolongations in the lag phase (p<0.01 vs. apixaban alone) and enhancing the maximum thrombin peak observed during TG (p<0.05 vs. apixaban alone). PCCs shortened the lag phase and enhanced TG initially altered by apixaban, though differences did not achieve the levels of statistical significance.

**Table 1:** Modifications in thrombin generation initiated in plasma with phospholipids and tissue factor.

RCL reagent	Lag phase (min)	Thrombin Peak (nM)	Time Peak (min)
CON	15.5±2.3	349.7±30.0	24.2±4.1
APIX	34.3±5.5**	75.8±23.0**	42.2±8.9
APIX + rFVIIa	15.4±1.9 ##	159.8±41.1 #	34.2±6.6
APIX + aPCC	14.2±1.1 ##	221.1±50.1#	37.1±5.0
APIX + PCC	25.0±4.5	169.4±7.0	42.8±8.7

Thrombin generation was initiated with TECHNOTHROMBIN^®^ RC Low (RCL) reagent containing phospholipids and tissue factor. APIX =  Apixaban 200 ng/mL; rFVIIa =  Novoseven® 270 µg/kg; aPCC =  Feiba® 75 U/kg; PCC =  Beriplex® 50 IU/kg. Values from 10 independent experiments, expressed as mean ± SEM, **: P<0.01 vs. CON; ##: P<0.01 vs. APIX.

Effects of apixaban in TG initiated by phospholipids (RD reagent) were less evident (Figure 1b and Table 2). Apixaban treatment caused a moderate prolongation in the time to reach a peak concentration of thrombin (10.4±1.1 min vs. 8.5±0.8 min in control studies, p<0.05), but did not cause significant alterations in the lag time or maximum thrombin peak. Addition of aPCCs and PCCs to plasma from blood anticoagulated with apixaban normalized the prolongation in the time to reach a peak in TG after apixaban and significantly enhanced the maximum thrombin peak with values that doubled those observed after apixaban (999.3±33.2 nM and 979.9±30.7 nM; p<0.01 vs. 474.1±13.6 nM in apixaban treated samples). Recombinant FVIIa did not show a relevant influence in the kinetics of TG previously altered by apixaban.

**Table 2:** Modifications in thrombin generation initiated in plasma with phospholipids micelles.

RD reagent	Lag phase (min)	Thrombin Peak (nM)	Time Peak (min)
CON	3.9±0.4	484.9±15.2	8.5±0.8
APIX	4.3±0.4	474.1±13.6	10.4±1.1**
APIX + rFVIIa	4.2±0.4	457.1±14.4	8.6±1.1##
APIX + aPCC	4.8±0.4	999.3±33.2##	9.3±1.1#
APIX + PCC	4.2±0.5	979.9±30.7##	9.4±0.8

Thrombin generation was initiated with TECHNOTHROMBIN^®^ RD reagent containing phospholipids. APIX =  Apixaban 200 ng/mL; rFVIIa =  Novoseven® 270 µg/kg; aPCC =  Feiba® 75 U/kg; PCC =  Beriplex® 50 IU/kg. Values from 10 independent experiments, expressed as mean ± SEM; * P<0.05 vs. CON, **: P<0.01 vs. CON; # P<0.05 vs. APIX ##: P<0.01 vs. APIX.

### Modifications in viscoelastic properties of clots

**Figure 2:**
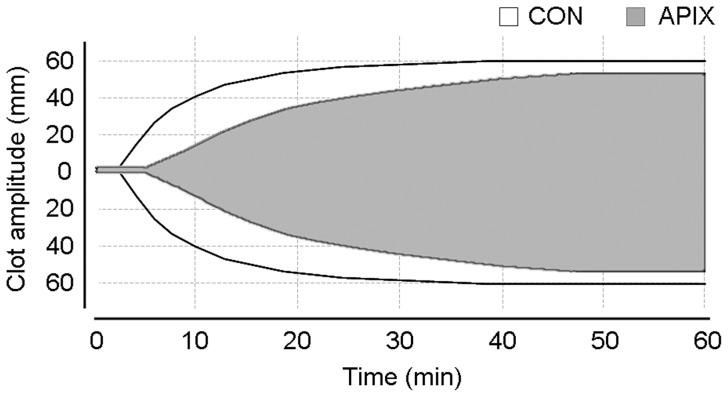
Effect of apixaban on the thromboelastometric properties of clot formation. Representative thromboelastograms generated in control (CON) and apixaban treated (APIX) blood samples. Studies were performed in recalcified citrated blood activated by exTEM reagent as indicated in the material and methods section. Apixaban 200 ng/mL prolonged parameters related to clot formation (clotting time = CT and clot formation time = CFT) with minimal impact on maximum clot firmness (MCF). Refer to Table 3 for detailed statistics.

As illustrated in Figure 2, apixaban modified the dynamics of clot formation in ROTEM experiments, but had little impact on the maximum clot firmness (MCF). As summarized in Table 3, apixaban caused a statistically significant prolongation of CT (357.9±52.8 sec vs. 165.6±19.7 sec in control studies; p<0.01) and CFT (923.8±3 sec vs. 262.9±39.4 sec in control studies). Addition of rFVIIa or aPCCs significantly reduced the prolongation in CT caused by apixaban (p<001 for both concentrates vs. treatment with apixaban alone). CTs achieved after addition of rFVIIa or aPCCs to apixaban anticoagulated blood (95.1±15.9 sec and 102.0±9.8 sec, respectively) were below those observed in control experiments (165.6±19.7 sec). Corrections of alterations induced by apixaban in ROTEM parameters were less evident with PCCs.

**Table 3:** Effects of apixaban on the thromboelastometric parameters during clot formation.

EXTEM	CT (s)	CFT (s)	MCF (mm)
CON	165.6±19.7	262.9±39.4	53.0±2.2
APIX	357.9±52.8**	923.8±353.9	40.2±7.7
APIX + rFVIIa	95.1±15.9##	140.1±33.9#	62.3±2.1#
APIX + aPCC	102.0±9.8##	116.1±12.5#	62.7±0.8#
APIX + PCC	326.2±86.3	430.7±117.6	56.9±1.6#

Modifications in clotting time (CT), clot formation time (CFT), and maximum clot firmness (MCF) assessed in ROTEM. Studies were performed in recalcified citrated blood activated by exTEM as indicated in the material and methods section. APIX =  Apixaban 200 ng/mL; rFVIIa =  Novoseven® 270 µg/kg; aPCC =  Feiba® 75 U/kg; PCC =  Beriplex® 50 IU/kg. Values from 10 independent experiments, expressed as mean ± SEM, * P<0.05 vs. CON, **: P<0.01 vs. CON; # P<0.05 vs. APIX ##: P<0.01 vs. APIX.

Modifications in CFT after reversal therapies followed the tendencies mentioned for CT, with differences in CFT after rFVIIa and aPCCs reaching levels of significance at p<0.05. All the concentrates tested, rFVIIa, aPCCs and PCCs, moderately enhanced MCF with respect to corresponding values in apixaban anticoagulated samples (62.3±2.1 mm, 62.7±0.8 mm, and 56.9±1.6 mm respectively, vs. 40.2±7.7 mm after apixaban; p<0.05).

### Perfusion Studies

**Figure 3:**
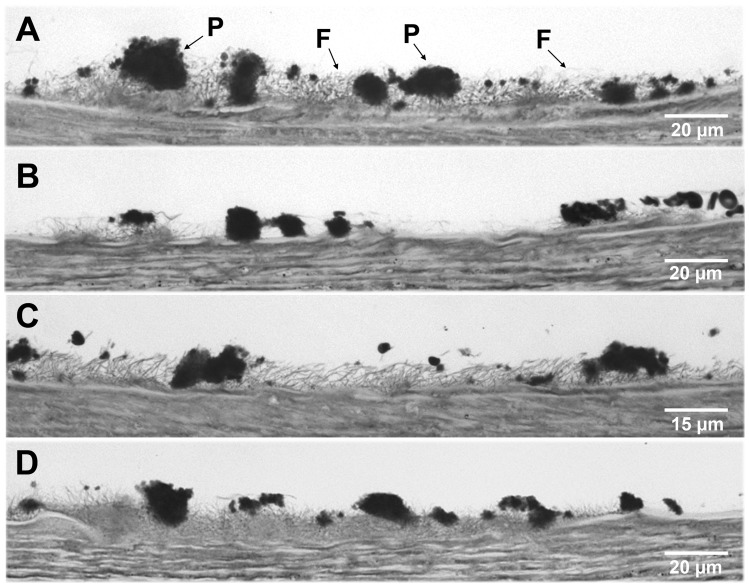
Effect of apixaban on platelet and fibrin interactions with damaged vessels exposed to flowing blood. Light microscopy images showing platelets and fibrin interactions on cross-sections of the perfused vessels. Perfusion studies with recalcified blood were performed at a shear rate of 600 s^−1^ with flowing blood for 10 min. Representative micrographs of (**A**) control studies and (**B**) blood incubated with apixaban 200 ng/mL. (**B**) Apixaban caused a reduction in fibrin formation and platelet interactions with the damaged vessels. The size of platelet aggregates was apparently decreased in samples exposed to apixaban. (**C**) Activated and non activated prothrombin complex concentrates, aPCCs and PCCs, restored levels of fibrin deposited on the subendothelium. (**D**) Recombinant factor VIIa (rFVIIa) restored fibrin deposition and improved platelet interactions with the damaged vessel. Images are representative of 10 independent experiments. P = Platelet aggregates; F =  Fibrin. Refer to Table 4 for numerical data and statistical comparisons.

Apixaban reduced platelet and fibrin components of hemostasis in perfusion studies (Figure 3 and Table 4). Results of the morphometric evaluations performed on damaged vessels perfused with blood incubated with apixaban revealed statistically significant reductions in: the percentage of the vessel covered by platelets (% CS: 12.1±1.8 vs. 20.9±3.0 in control experiments; p<0.01), and fibrin (% F: 17.2±4.1 vs. 52.2±11.6 in control experiments; p<0.05). Apixaban interfered with the formation of large platelet aggregates (% Agg) causing statistically significant reductions (% Agg: 8.5±1.3 vs. 15.0±2.6 in controls studies, p<0.01). The cross sectional average area of fibrin masses deposited on the vessel surface was markedly reduced after apixaban (p<0.05 vs. control experiments).

**Table 4:** Modifications in platelet and fibrin components of hemostatic plugs formed on perfused vascular surfaces.

Perfusion	% CS	% Agg	% F	F Area (µm^2^)
CON	20.9±3.0	15.0±2.6	52.2±11.6	275.1±96.3
APIX	12.1±1.8**	8.5±1.3**	17.2±4.1**	53.2±10.8*
APIX + rFVIIa	17.9±2.7#	14.4±2.7#	46.2±8.6##	236.6±77.5#
APIX + aPCC	15.5±2.5	10.3±1.8	45.9±9.4##	218.7±50.4##
APIX + PCC	14.1±2.4	11.0±2.0	53.0±9.0##	249.6±87.0#

Morphometric evaluation of platelets and fibrin interactions with damaged vascular surfaces in perfusion studies with citrated whole blood. Blood was recalcified and perfused at a shear rate of 600 s^−1^. Morphometric results are expressed as: percentage of covered surface by platelets (% CS), by large platelet aggregates (% Agg), or by fibrin (% F), and average area of fibrin masses deposited (F Area). APIX =  Apixaban 200 ng/mL; rFVIIa =  Novoseven® 270 µg/kg; aPCC =  Feiba® 75 U/kg; PCC =  Beriplex® 50 IU/kg. Values from 10 independent experiments, expressed as mean ± SEM; * P<0.05 vs. CON; **: P<0.01 vs. CON; # P<0.05 vs. APIX; ## P<0.01 vs. APIX.

PCCs and aPCCs did not show a favorable action at improving the reduction of platelet interactions with the subendothelium observed after apixaban. Recombinant FVIIa partially counteracted the reductions in platelet interactions induced by apixaban (% CS: 17.9±2.7 vs. 12.1±1.8, p<0.05; and % Agg: 14.4±2.7 vs. 8.5±1.3, p<0.05; in both cases vs. apixaban treated blood). Addition of rFVIIa, aPCCs or PCCs restored levels of fibrin deposited on the subendothelium, initially reduced by apixaban, to levels observed in control samples (p<0.01 vs. apixaban treated blood). The ability of rFVIIa, aPCCs and PCCs to improve fibrin generation was also evidenced by their ability to increase the cross sectional areas of fibrin masses initially reduced by apixaban (p<0.05 for rFVIIa and PCCs; and p<0.01 for aPCCs).

## Discussion

In the present study we have applied a series of experimental approaches to human blood to characterize the effects of apixaban on platelet and coagulation parameters. Apixaban interfered with thrombin generation, altered viscoelastic parameters during clot formation and reduced platelet and fibrin formation on damaged vessels. Alterations in coagulation parameters induced by apixaban were variably compensated or even reversed by the different coagulation factor concentrates used in our studies, though responses to these concentrates were not homogeneous throughout all tests.

Reversal of the alterations in hemostasis caused by the new oral anticoagulant agents is a subject of interest, with most of the evidence published previously resulting from experimental animals or derived from anecdotal cases [1], [8], [15], [30], [31]. Several studies have applied *ex vivo* and *in vivo* approaches to investigate the reversal of the anticoagulant action of dabigatran and rivaroxaban, using coagulation factor concentrates already used for the control of the anticogulant action of classic vitamin K (Vit K) antagonists, with successful results [13], [16], [17]. Evaluation of the kinetics of thrombin generation (TG) has been used to assess the effects of reversal strategies on the alterations in coagulation caused by new oral anticoagulants [16], [17], [21]. Our studies with a TG test based on the calibrated automated thrombogram (CAT) assay, revealed important variations between results that were clearly related to the activating reagents. One of the reagents (RCL) contains a low concentration of phospholipids micelles and recombinant human TF, while the other (RD) is composed only of phospholipid micelles without TF. Effects of apixaban were very obvious when TG was activated by TF and phospholipids through the extrinsic pathway and less evident when using the phospholipid based activator triggering the intrinsic pathway of coagulation. Interestingly, the effectiveness of reversal therapies at restoring TG differed strongly depending on the activator with very mild responses to PCCs with the TF containing reagent, and with explosive responses to PCCs and aPCCs with in the phospholipid-based activator. Which TG tests reflects more precisely the impact of the new oral an anticoagulant, how modifications of the different parameters relate to a potential bleeding risk and whether modification in these parameters have a predictive value in the correction of excessive anticoagulation is an object of debate [18], [32], [33]. In a recent study, Dinkelaar and cols [34] investigated the applicability of PCCs to the reversal of rivaroxaban induced alterations on thrombin generation. These authors concluded that responses to different TG tests were clearly dependent on the assay conditions and the amount of PCC required for normalization of TG depended on the concentration of TF and the presence of phospholipids. Our results would further exemplify the relevance of assay conditions for TG tests and may hel reconciling discrepancies between results in TG assays and correction of experimental bleeding in animal models [18].

Apixaban altered the dynamics of clot formation in ROTEM assays as confirmed by significantly prolonged clotting times and minimally affected clot firmness. All the concentrates tested in our studies reduced the prolongation in clotting times and significantly enhanced the mean clot firmness in this assay. Our present results suggest that ROTEM could be applied for a rapid assessment of the effects of excessive anticoagulation caused by elevated doses of apixaban and tentatively for the assessment of its reversal by different factor concentrates.

Current coagulation tests, including TG and ROTEM assays, evaluate specific coagulation pathways or endpoints, but do not provide information on the coordinated interactions between platelets and fibrin that ultimately warrants effective control of hemostasis. The previous rationale could explain why corrections of laboratory parameters, including TG, observed after factor concentrates do not correspond with control of bleeding symptoms observed by other investigators [18], [35]. The problem with many of the currently available coagulation assays is that they often disregard rheological conditions that regulate cellular and plasma interactions with damaged vascular surfaces resulting in the initiation and consolidation of hemostasis.

In an attempt to circumvent the potential limitations of current coagulation tests, our investigational approach included evaluation of thrombus formation on damaged vascular surfaces in studies performed with blood maintained under specific flow conditions. An advantage of this methodology is that studies can be performed *in vitro* or *ex vivo* spiking blood with therapeutic agents without exposing patients or volunteers to additional risks. Studies in perfusion devices have helped to improve basic and clinical knowledge on the effectiveness of hemostatic strategies in transfusion medicine [36], including effects of platelet concentrates [37] and rFVIIa [38], [39]. Our studies with this technique reveal that apixaban significantly reduced both, platelet and fibrin deposition on perfused vessels. Impairments in fibrin formation were effectively corrected by the different concentrates with minor differences among them. Interestingly, only rFVIIa was able to fully normalize levels of platelet deposition. Previous studies from our laboratories recognized that rFVIIa could locally enhance TG and improve the recruitment of platelets into forming aggregates [28].

Recommendation on the more adequate strategy for reversal of the anticoagulant effects of apixaban should be based on a balanced evaluation of severity of the bleeding situation, the efficacy of the concentrates chosen, and the possible side effects derived from our reversal intervention. Despite the fact that rFVIIa showed the most effective action at correcting platelet and fibrin formation in perfusion studies, there is concern regarding is potential thrombogenicity when this recombinant factor is used out of the approved indications [40]. Activated PCCs have already shown an enhanced risk of thrombosis when used in patients with hemophilia [41] and this risk would be expected to increase if applied to patients with an underlying prothrombotic condition. In our study, four-factor PCCs showed a clear potential reversal in the majority of the tests including perfusion studies. There is wide experience with the use four-factor PCCs in the reversal of the anticoagulant effect of Vit K antagonists and rates of thrombotic complications seem to be acceptable [42]. Thus, PCCs appear as the first line reversal agent for apixaban until other most specific agents demonstrate efficacy and safety in the clinical setting.

In summary, we demonstrate that alterations in hemostasis caused by apixaban are variably compensated or even reversed by the different factor concentrates tested in our studies. Effects of these concentrates at reversing alterations of platelets and fibrin formation were assessed in experiments with blood flowing through damaged vessels an experimental setting that resembles the bleeding situation. Further experimental studies would be required to find appropriate doses for the different concentrates that could be evaluated in specific clinical trials.

## Supporting Information

Methods S1 Perfusion technique. Studies in perfusion devices have been useful to improve basic and clinical knowledge on the effectiveness of hemostatic strategies in transfusion medicine, including effects of platelet concentrates and rFVIIa. Aliquots of blood are perfused through annular chambers exposing damaged vascular segments, as thrombogenic substrata, at a fixed shear-rate. This supplemental file provides visual information on this methodology, from the extraction of the rabbit aorta to be used as thrombogenic substrata, to the final morphometric analysis on cross-sections of the perfused vessels.
(EXE)
